# Supplementary material for: Modeling glioblastoma heterogeneity as a dynamic network of cell states
Source: Mol Syst Biol. 2021 Sep 16;17(9):e10105. doi: 10.15252/msb.202010105 (PMC8444284; doi:10.15252/msb.202010105)
Supplement: Supplementary file 5 — Source Data for Figure 3 [file MSB-17-e10105-s001.zip › Figure3A_sourcedata/GSEA_3065/nosigdb_state6.GseaPreranked.1622718443993/DOROSZKO_U3013MG_CLUSTER_2.html]

Details for gene set DOROSZKO\_U3013MG\_CLUSTER\_2[GSEA]

|  || Dataset | state6 |
| Phenotype | NoPhenotypeAvailable |
| Upregulated in class | na\_neg |
| GeneSet | DOROSZKO\_U3013MG\_CLUSTER\_2 |
| Enrichment Score (ES) | -0.543624 |
| Normalized Enrichment Score (NES) | -2.3996718 |
| Nominal p-value | 0.0 |
| FDR q-value | 0.0 |
| FWER p-Value | 0.0 |
Table: GSEA Results Summary

  

Fig 1: Enrichment plot: DOROSZKO\_U3013MG\_CLUSTER\_2      
 Profile of the Running ES Score & Positions of GeneSet Members on the Rank Ordered List

  

| PROBE | GENE SYMBOL | GENE\_TITLE | RANK IN GENE LIST | RANK METRIC SCORE | RUNNING ES | CORE ENRICHMENT || 1 | HDGF |  |  | 247 | 0.162 | -0.0192 | No |
| 2 | TMSB15A |  |  | 253 | 0.161 | -0.0102 | No |
| 3 | NEURL1B |  |  | 359 | 0.142 | -0.0140 | No |
| 4 | YWHAH |  |  | 449 | 0.128 | -0.0168 | No |
| 5 | PSMD10 |  |  | 504 | 0.123 | -0.0159 | No |
| 6 | KHDRBS1 |  |  | 525 | 0.120 | -0.0111 | No |
| 7 | SKA2 |  |  | 568 | 0.116 | -0.0091 | No |
| 8 | BCL7C |  |  | 743 | 0.102 | -0.0233 | No |
| 9 | SAC3D1 |  |  | 860 | 0.094 | -0.0313 | No |
| 10 | EZH2 |  |  | 940 | 0.089 | -0.0352 | No |
| 11 | MYBL2 |  |  | 991 | 0.086 | -0.0359 | No |
| 12 | MT2A |  |  | 1097 | 0.081 | -0.0434 | No |
| 13 | CENPH |  |  | 1340 | 0.070 | -0.0674 | No |
| 14 | SAP30 |  |  | 1409 | 0.067 | -0.0714 | No |
| 15 | PPP2CA |  |  | 1574 | 0.061 | -0.0868 | No |
| 16 | USP1 |  |  | 1712 | 0.056 | -0.0995 | No |
| 17 | RHEB |  |  | 1873 | 0.051 | -0.1150 | No |
| 18 | BTG3 |  |  | 2012 | 0.047 | -0.1283 | No |
| 19 | JPT1 |  |  | 2094 | 0.045 | -0.1351 | No |
| 20 | CENPA |  |  | 2199 | 0.042 | -0.1447 | No |
| 21 | TTK |  |  | 2262 | 0.040 | -0.1496 | No |
| 22 | CBX5 |  |  | 2269 | 0.039 | -0.1480 | No |
| 23 | HJURP |  |  | 2286 | 0.039 | -0.1475 | No |
| 24 | PTTG1 |  |  | 2304 | 0.038 | -0.1472 | No |
| 25 | CENPQ |  |  | 2324 | 0.038 | -0.1472 | No |
| 26 | MND1 |  |  | 2472 | 0.034 | -0.1623 | No |
| 27 | RPA3 |  |  | 2563 | 0.032 | -0.1709 | No |
| 28 | ANP32E |  |  | 2570 | 0.032 | -0.1697 | No |
| 29 | POC1A |  |  | 2667 | 0.030 | -0.1792 | No |
| 30 | CCP110 |  |  | 2781 | 0.027 | -0.1907 | No |
| 31 | HIRIP3 |  |  | 2798 | 0.027 | -0.1910 | No |
| 32 | CBR3 |  |  | 2908 | 0.024 | -0.2023 | No |
| 33 | TMPO |  |  | 2978 | 0.022 | -0.2090 | No |
| 34 | POLR2A |  |  | 2984 | 0.022 | -0.2082 | No |
| 35 | CKS1B |  |  | 2985 | 0.022 | -0.2069 | No |
| 36 | NSD2 |  |  | 3106 | 0.020 | -0.2197 | No |
| 37 | PLK1 |  |  | 3116 | 0.020 | -0.2196 | No |
| 38 | MASTL |  |  | 3217 | 0.017 | -0.2302 | No |
| 39 | DTYMK |  |  | 3271 | 0.016 | -0.2354 | No |
| 40 | HMGB3 |  |  | 3377 | 0.014 | -0.2469 | No |
| 41 | PSRC1 |  |  | 3406 | 0.013 | -0.2494 | No |
| 42 | RACGAP1 |  |  | 3432 | 0.012 | -0.2515 | No |
| 43 | PHF19 |  |  | 3654 | 0.008 | -0.2768 | No |
| 44 | ALYREF |  |  | 3755 | 0.006 | -0.2881 | No |
| 45 | HP1BP3 |  |  | 3877 | 0.003 | -0.3020 | No |
| 46 | CDC25B |  |  | 3887 | 0.003 | -0.3029 | No |
| 47 | CENPW |  |  | 3936 | 0.002 | -0.3084 | No |
| 48 | CDC20 |  |  | 3996 | 0.001 | -0.3152 | No |
| 49 | KRBOX1 |  |  | 4081 | -0.001 | -0.3249 | No |
| 50 | PARPBP |  |  | 4139 | -0.002 | -0.3315 | No |
| 51 | CKLF |  |  | 4263 | -0.004 | -0.3455 | No |
| 52 | G2E3 |  |  | 4365 | -0.006 | -0.3569 | No |
| 53 | SOGA1 |  |  | 4484 | -0.008 | -0.3701 | No |
| 54 | ZWILCH |  |  | 4527 | -0.009 | -0.3745 | No |
| 55 | CDCA5 |  |  | 4577 | -0.010 | -0.3796 | No |
| 56 | CDKN2D |  |  | 4667 | -0.012 | -0.3892 | No |
| 57 | CCNB2 |  |  | 4736 | -0.013 | -0.3964 | No |
| 58 | DDX39A |  |  | 4809 | -0.014 | -0.4039 | No |
| 59 | LMNB2 |  |  | 4854 | -0.016 | -0.4081 | No |
| 60 | ANP32B |  |  | 4891 | -0.016 | -0.4113 | No |
| 61 | ASF1B |  |  | 4895 | -0.016 | -0.4107 | No |
| 62 | KIF14 |  |  | 4913 | -0.017 | -0.4117 | No |
| 63 | RTKN2 |  |  | 4914 | -0.017 | -0.4107 | No |
| 64 | CENPM |  |  | 5089 | -0.020 | -0.4297 | No |
| 65 | C21orf58 |  |  | 5096 | -0.020 | -0.4292 | No |
| 66 | GTSE1 |  |  | 5195 | -0.022 | -0.4393 | No |
| 67 | HNRNPH3 |  |  | 5348 | -0.025 | -0.4555 | No |
| 68 | BUB1 |  |  | 5377 | -0.026 | -0.4573 | No |
| 69 | H2AFX |  |  | 5451 | -0.027 | -0.4641 | No |
| 70 | CDCA8 |  |  | 5535 | -0.029 | -0.4721 | No |
| 71 | LMNB1 |  |  | 5545 | -0.029 | -0.4714 | No |
| 72 | ECT2 |  |  | 5560 | -0.030 | -0.4713 | No |
| 73 | UACA |  |  | 5586 | -0.030 | -0.4724 | No |
| 74 | CAPS |  |  | 5598 | -0.030 | -0.4719 | No |
| 75 | BUB3 |  |  | 5807 | -0.034 | -0.4940 | No |
| 76 | MNS1 |  |  | 5869 | -0.035 | -0.4991 | No |
| 77 | KNSTRN |  |  | 5875 | -0.036 | -0.4975 | No |
| 78 | MAGOH |  |  | 5994 | -0.038 | -0.5090 | No |
| 79 | NUDT1 |  |  | 6071 | -0.040 | -0.5155 | No |
| 80 | RFC3 |  |  | 6078 | -0.040 | -0.5139 | No |
| 81 | FAM111A |  |  | 6097 | -0.040 | -0.5136 | No |
| 82 | SPC24 |  |  | 6135 | -0.041 | -0.5155 | No |
| 83 | SPAG5 |  |  | 6146 | -0.041 | -0.5142 | No |
| 84 | CDCA2 |  |  | 6175 | -0.042 | -0.5150 | No |
| 85 | CENPN |  |  | 6194 | -0.042 | -0.5146 | No |
| 86 | AURKB |  |  | 6215 | -0.043 | -0.5143 | No |
| 87 | DNAJC9 |  |  | 6230 | -0.043 | -0.5134 | No |
| 88 | CDKN3 |  |  | 6291 | -0.044 | -0.5178 | No |
| 89 | PCLAF |  |  | 6365 | -0.046 | -0.5235 | No |
| 90 | BCL2L12 |  |  | 6401 | -0.047 | -0.5248 | No |
| 91 | FANCI |  |  | 6473 | -0.049 | -0.5302 | No |
| 92 | SPDL1 |  |  | 6573 | -0.052 | -0.5386 | No |
| 93 | PTGES3 |  |  | 6602 | -0.053 | -0.5388 | No |
| 94 | HMMR |  |  | 6640 | -0.054 | -0.5399 | No |
| 95 | FAM83D |  |  | 6673 | -0.055 | -0.5404 | Yes |
| 96 | FOXM1 |  |  | 6684 | -0.055 | -0.5383 | Yes |
| 97 | TEDC1 |  |  | 6696 | -0.056 | -0.5363 | Yes |
| 98 | TROAP |  |  | 6707 | -0.056 | -0.5341 | Yes |
| 99 | BUB1B |  |  | 6717 | -0.056 | -0.5319 | Yes |
| 100 | PSMC3IP |  |  | 6749 | -0.057 | -0.5321 | Yes |
| 101 | MKI67 |  |  | 6750 | -0.057 | -0.5287 | Yes |
| 102 | CDC27 |  |  | 6787 | -0.058 | -0.5294 | Yes |
| 103 | MXD3 |  |  | 6808 | -0.059 | -0.5283 | Yes |
| 104 | CIP2A |  |  | 6905 | -0.062 | -0.5358 | Yes |
| 105 | PRC1 |  |  | 6918 | -0.062 | -0.5335 | Yes |
| 106 | GPSM2 |  |  | 6953 | -0.063 | -0.5337 | Yes |
| 107 | RAD21 |  |  | 6984 | -0.064 | -0.5334 | Yes |
| 108 | TACC3 |  |  | 7022 | -0.066 | -0.5338 | Yes |
| 109 | BIRC5 |  |  | 7024 | -0.066 | -0.5301 | Yes |
| 110 | CKAP2L |  |  | 7065 | -0.067 | -0.5308 | Yes |
| 111 | SHCBP1 |  |  | 7076 | -0.067 | -0.5280 | Yes |
| 112 | KIF22 |  |  | 7098 | -0.068 | -0.5264 | Yes |
| 113 | REEP4 |  |  | 7127 | -0.069 | -0.5256 | Yes |
| 114 | ACTL6A |  |  | 7183 | -0.070 | -0.5279 | Yes |
| 115 | CDK5RAP2 |  |  | 7213 | -0.071 | -0.5271 | Yes |
| 116 | FEN1 |  |  | 7218 | -0.071 | -0.5233 | Yes |
| 117 | RAD51AP1 |  |  | 7236 | -0.072 | -0.5210 | Yes |
| 118 | MIS18BP1 |  |  | 7253 | -0.073 | -0.5186 | Yes |
| 119 | TRIP13 |  |  | 7291 | -0.074 | -0.5185 | Yes |
| 120 | ZWINT |  |  | 7370 | -0.077 | -0.5230 | Yes |
| 121 | TOP2A |  |  | 7430 | -0.079 | -0.5253 | Yes |
| 122 | NCAPH |  |  | 7438 | -0.079 | -0.5214 | Yes |
| 123 | RRM1 |  |  | 7491 | -0.081 | -0.5227 | Yes |
| 124 | TK1 |  |  | 7499 | -0.081 | -0.5187 | Yes |
| 125 | SPC25 |  |  | 7522 | -0.082 | -0.5164 | Yes |
| 126 | PIMREG |  |  | 7526 | -0.083 | -0.5119 | Yes |
| 127 | LSM8 |  |  | 7596 | -0.086 | -0.5148 | Yes |
| 128 | CCDC34 |  |  | 7609 | -0.087 | -0.5111 | Yes |
| 129 | KIF11 |  |  | 7619 | -0.087 | -0.5070 | Yes |
| 130 | CDCA3 |  |  | 7631 | -0.087 | -0.5032 | Yes |
| 131 | SGO2 |  |  | 7632 | -0.087 | -0.4980 | Yes |
| 132 | DEPDC1 |  |  | 7637 | -0.087 | -0.4933 | Yes |
| 133 | DBF4 |  |  | 7639 | -0.088 | -0.4883 | Yes |
| 134 | HIST1H4C |  |  | 7641 | -0.088 | -0.4832 | Yes |
| 135 | GMNN |  |  | 7651 | -0.088 | -0.4790 | Yes |
| 136 | LSM5 |  |  | 7653 | -0.088 | -0.4739 | Yes |
| 137 | EXOSC8 |  |  | 7671 | -0.089 | -0.4706 | Yes |
| 138 | AURKA |  |  | 7700 | -0.091 | -0.4685 | Yes |
| 139 | NDC80 |  |  | 7734 | -0.093 | -0.4669 | Yes |
| 140 | CDKN2C |  |  | 7743 | -0.093 | -0.4623 | Yes |
| 141 | ESCO2 |  |  | 7778 | -0.094 | -0.4607 | Yes |
| 142 | KNL1 |  |  | 7810 | -0.096 | -0.4586 | Yes |
| 143 | UBE2S |  |  | 7818 | -0.097 | -0.4537 | Yes |
| 144 | PGP |  |  | 7898 | -0.101 | -0.4570 | Yes |
| 145 | PBK |  |  | 7905 | -0.101 | -0.4517 | Yes |
| 146 | NUF2 |  |  | 7936 | -0.103 | -0.4491 | Yes |
| 147 | ATAD5 |  |  | 7944 | -0.104 | -0.4438 | Yes |
| 148 | CCNB1 |  |  | 7958 | -0.104 | -0.4391 | Yes |
| 149 | ITGB3BP |  |  | 7959 | -0.104 | -0.4330 | Yes |
| 150 | KIFC1 |  |  | 7968 | -0.105 | -0.4277 | Yes |
| 151 | CEP55 |  |  | 7982 | -0.106 | -0.4230 | Yes |
| 152 | RRM2 |  |  | 8001 | -0.107 | -0.4187 | Yes |
| 153 | CACYBP |  |  | 8012 | -0.108 | -0.4135 | Yes |
| 154 | KPNA2 |  |  | 8045 | -0.110 | -0.4107 | Yes |
| 155 | WEE1 |  |  | 8054 | -0.111 | -0.4051 | Yes |
| 156 | CKS2 |  |  | 8077 | -0.112 | -0.4011 | Yes |
| 157 | NCAPD3 |  |  | 8089 | -0.113 | -0.3957 | Yes |
| 158 | POP7 |  |  | 8147 | -0.117 | -0.3954 | Yes |
| 159 | ANLN |  |  | 8181 | -0.120 | -0.3922 | Yes |
| 160 | XPO1 |  |  | 8216 | -0.123 | -0.3889 | Yes |
| 161 | CCNA2 |  |  | 8227 | -0.124 | -0.3827 | Yes |
| 162 | KIF15 |  |  | 8228 | -0.124 | -0.3754 | Yes |
| 163 | LBR |  |  | 8242 | -0.125 | -0.3695 | Yes |
| 164 | TYMS |  |  | 8287 | -0.131 | -0.3669 | Yes |
| 165 | KIF4A |  |  | 8300 | -0.132 | -0.3605 | Yes |
| 166 | DIAPH3 |  |  | 8315 | -0.134 | -0.3542 | Yes |
| 167 | KIF2C |  |  | 8328 | -0.135 | -0.3476 | Yes |
| 168 | CKAP2 |  |  | 8330 | -0.136 | -0.3397 | Yes |
| 169 | CDK1 |  |  | 8351 | -0.139 | -0.3338 | Yes |
| 170 | ATAD2 |  |  | 8359 | -0.141 | -0.3263 | Yes |
| 171 | TUBA1C |  |  | 8365 | -0.142 | -0.3185 | Yes |
| 172 | NCAPD2 |  |  | 8367 | -0.142 | -0.3103 | Yes |
| 173 | TPX2 |  |  | 8370 | -0.143 | -0.3020 | Yes |
| 174 | HMGB2 |  |  | 8388 | -0.145 | -0.2955 | Yes |
| 175 | NCAPG |  |  | 8390 | -0.145 | -0.2870 | Yes |
| 176 | PHIP |  |  | 8391 | -0.145 | -0.2784 | Yes |
| 177 | CENPU |  |  | 8403 | -0.147 | -0.2710 | Yes |
| 178 | MAD2L1 |  |  | 8427 | -0.150 | -0.2649 | Yes |
| 179 | MELK |  |  | 8437 | -0.150 | -0.2570 | Yes |
| 180 | CLSPN |  |  | 8440 | -0.151 | -0.2483 | Yes |
| 181 | DLGAP5 |  |  | 8447 | -0.152 | -0.2401 | Yes |
| 182 | KIF23 |  |  | 8465 | -0.154 | -0.2329 | Yes |
| 183 | ORC6 |  |  | 8482 | -0.157 | -0.2255 | Yes |
| 184 | UBE2T |  |  | 8489 | -0.159 | -0.2168 | Yes |
| 185 | ARHGAP11A |  |  | 8513 | -0.164 | -0.2098 | Yes |
| 186 | NUSAP1 |  |  | 8523 | -0.167 | -0.2010 | Yes |
| 187 | CKAP5 |  |  | 8532 | -0.169 | -0.1920 | Yes |
| 188 | TUBB4B |  |  | 8554 | -0.173 | -0.1842 | Yes |
| 189 | SGO1 |  |  | 8571 | -0.178 | -0.1755 | Yes |
| 190 | KIF20B |  |  | 8576 | -0.180 | -0.1653 | Yes |
| 191 | ILF2 |  |  | 8579 | -0.181 | -0.1549 | Yes |
| 192 | PRR11 |  |  | 8580 | -0.182 | -0.1441 | Yes |
| 193 | NUCB2 |  |  | 8584 | -0.184 | -0.1336 | Yes |
| 194 | DHFR |  |  | 8590 | -0.186 | -0.1232 | Yes |
| 195 | ASPM |  |  | 8657 | -0.215 | -0.1181 | Yes |
| 196 | SMC2 |  |  | 8658 | -0.216 | -0.1054 | Yes |
| 197 | BRCA1 |  |  | 8659 | -0.217 | -0.0926 | Yes |
| 198 | CENPK |  |  | 8666 | -0.220 | -0.0803 | Yes |
| 199 | GGH |  |  | 8702 | -0.239 | -0.0702 | Yes |
| 200 | CENPF |  |  | 8703 | -0.240 | -0.0560 | Yes |
| 201 | SYPL1 |  |  | 8724 | -0.261 | -0.0429 | Yes |
| 202 | CENPE |  |  | 8734 | -0.270 | -0.0280 | Yes |
| 203 | UBE2C |  |  | 8748 | -0.286 | -0.0127 | Yes |
| 204 | SMC4 |  |  | 8764 | -0.307 | 0.0037 | Yes |
Table: GSEA details [plain text format]

  

Fig 2: DOROSZKO\_U3013MG\_CLUSTER\_2: Random ES distribution      
 Gene set null distribution of ES for **DOROSZKO\_U3013MG\_CLUSTER\_2**
